# Highly Reversible Zn Anode Design Through Oriented ZnO(002) Facets

**DOI:** 10.1002/adma.202408908

**Published:** 2024-10-21

**Authors:** Chengwu Yang, Pattaraporn Woottapanit, Sining Geng, Kittima Lolupiman, Xinyu Zhang, Zhiyuan Zeng, Guanjie He, Jiaqian Qin

**Affiliations:** ^1^ Center of Excellence in Responsive Wearable Materials Metallurgy and Materials Science Research Institute Chulalongkorn University Bangkok 10330 Thailand; ^2^ State Key Laboratory of Metastable Materials Science and Technology Yanshan University Qinhuangdao 066004 P. R. China; ^3^ Department of Materials Science and Engineering and State Key Laboratory of Marine Pollution City University of Hong Kong 83 Tat Chee Avenue Kowloon Hong Kong 999077 P. R. China; ^4^ Shenzhen Research Institute City University of Hong Kong Shenzhen 518057 P. R. China; ^5^ Christopher Ingold Laboratory Department of Chemistry University College London London WC1H 0AJ UK

**Keywords:** hydrogen evolution suppression, zincophilicity, Zn anode, Zn ion batteries, ZnO (002) lattice plane

## Abstract

The practical implementation of aqueous Zn‐ion batteries presents formidable hurdles, including uncontrolled dendrite growth, water‐induced side reactions, suboptimal Zn metal utilization, and intricate Zn anode manufacturing. Here, large‐scale construction of a highly oriented ZnO(002) lattice plane on Zn anode (ZnO(002)@Zn) with thermodynamic inertia and kinetic zincophilicity is designed to address such problems. Both theoretical calculations and experiment results elucidate that the ZnO(002)@Zn possesses high Zn chemical affinity, hydrogen evolution reaction suppression, and dendrite‐free deposition ability due to the abundant lattice oxygen species in ZnO(002) and its low lattice mismatch with Zn(002). These features synergistically promote ion transport and enable homogeneous Zn deposition. Consequently, the ZnO(002)@Zn anode displays a stable and prolonged cycling lifespan exceeding 500 h even under a larger depth of discharge (85.6%) and realizes an impressive average Coulombic efficiency of 99.7%. Moreover, its efficacy is also evident in V_2_O_5_‐cathode coin cells and pouch cells with not only high discharge capacity but also exceptional cycling stability. This integrated approach presents a promising avenue for addressing the challenges associated with Zn metal anodes, thereby advancing the prospects of aqueous Zn‐ion battery technologies.

## Graphical abstract

ZnO(002) lattice plane with low lattice mismatch to Zn(002) endows fast ion diffusion, high Coulombic efficiency, and uniform Zn deposition, while ZnO(100) plane tends to exacerbate dendrite growth and provoke unwanted side reactions compared to bare Zn. Therefore, ZnO(002) protection layer on Zn realizes more stable performance for Zn‐ion batteries.

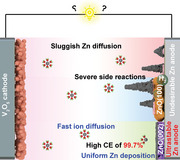

## 1 Introduction

Aqueous Zn ion batteries (AZIBs) stand poised as mighty candidates for large‐scale energy storage devices due to their inherent safety, cost‐effectiveness, and impressive theoretical specific capacity (820 mAh g^−1^).^[^ ^1^, ^2^, ^3^ ^]^ Nevertheless, AZIBs grapple with a pivotal challenge: the intensive Zn dendrite growth, resulting from the low zincophilicity of Zn metal, limited nucleation sites, and nonuniform Zn plating/stripping.^[^ ^4^, ^5^ ^]^ This phenomenon accelerates active Zn depletion and fosters internal short‐circuits, drastically curtailing battery lifespan and impeding practical applications.^[^ ^6^ ^]^ Additionally, the high thermodynamic activity of Zn metals, attributed to its low redox potential (−0.76 V vs standard hydrogen electrode), engenders detrimental parasitic side reactions, encompassing surface corrosion, hydrogen evolution reaction (HER), and the formation of undesirable by‐products, thus undermining Coulombic efficiency (CE) and precipitating rapid capacity decay.^[^ ^7^, ^8^ ^]^ Till now, many strategies have been pursued to fortify the stability of Zn anodes and extend their operational longevity, including the design of solid‐electrolyte interfaces (SEIs), electrolyte fine‐tuning, and separator modification.^[^ ^9^ ^]^ Even so, achieving uniform Zn deposition remains a formidable endeavor, especially under high depth of discharge (DOD, more than 80%).^[^ ^10^ ^]^ Hence, there exists an imperative and pressing need to effectively mitigate the intrinsic challenges associated with Zn anodes.

**Figure 1:**
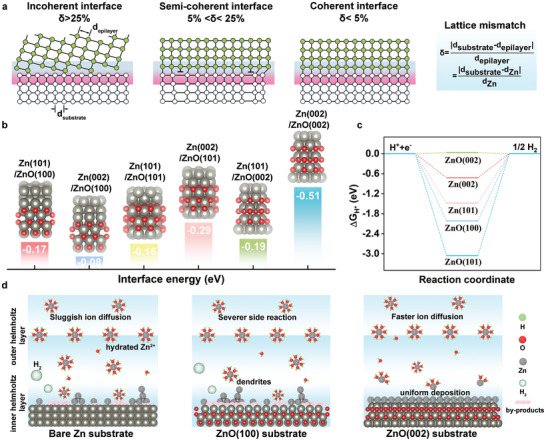
a) Interface structure models of Zn deposition on the substrates with incoherent, semi‐coherent, and coherent interfaces. Reproduced with permission.^[^ ^3^ ^]^ Copyright 2024, Springer Nature. b) Interface energy of Zn(101) and Zn(002) on ZnO(100), ZnO(101), and ZnO(002). c) The calculated ∆G_H*_ of H atom on different substrates. d) Schematic illustration of Zn deposition on the substrates.

Recently, numerous studies have highlighted the critical role of the preferential Zn(002) deposition in bolstering the endurance of Zn anodes during cycling.^[^ ^11^, ^12^ ^]^ Typically, the orientation of the electrodeposited Zn lattice plane exhibits a specific relationship with the substrate. Based on the classical interface theory, the lattice mismatch (δ) between deposited Zn and the substrate categorizes the phase interfaces into coherent (δ < 5%), semi‐coherent (5% < δ < 25%), and incoherent (δ > 25%) regions (**Figure** **1**a).^[^ ^13^ ^]^ A smaller lattice misfit signifies a stronger lattice orientation correlation between Zn and the substrate.^[^ ^14^ ^]^ Thus, the search and development of a suitable substrate with minimal lattice mismatch to Zn(002) are imperative. Presently, various substrates, including 2D materials and Zn‐based alloys, have been investigated.^[^ ^15^ ^]^ However, many of them exhibit high electronic conductivity and fail to isolate Zn metal anode from the aqueous electrolyte, consequently promoting electrochemical side reactions. Meanwhile, artificially engineered substrates that rely on intricate ex‐situ synthetic processes expose themselves to the risk of substrate delamination and exposure of fresh Zn, in turn leading to dendrite formation and increasing electronic resistance.^[^ ^16^ ^]^ What's more, a simple and low‐cost modification route for Zn anodes is necessary for the industrial manufacturing of AZIBs. Hence, spontaneous and large‐scale construction of a suitable substrate with solid adhesion, low conductivity, and minimal lattice mismatch on Zn electrodes holds immense promise for the development of stable AZIBs.

During the initial Zn plating/stripping process, electrochemical ZnO precipitates can form on the Zn surfaces due to water molecule decomposition near the Zn electrode, which provides some degree of protection against aqueous electrolyte erosion.^[^ ^17^ ^]^ However, as the cycle progresses, the ZnO accumulation will greatly increase the interfacial resistance and result in battery performance degradation. Interestingly, comparative analysis reveals that the (101) and (002) planes of ZnO have a low lattice mismatch with Zn(002) in contrast to the (100) plane (Figures S1 and S2, Supporting Information). By theoretical simulations and analysis in this work, it can be found that the ZnO(002) plane exhibits exceptional zincophilicity, high selectivity of Zn(002), and more favorable Gibbs free energy of hydrogen adsorption (∆G_H*_) than the ZnO(101), whereas the ZnO(100) fares considerably worse in these regards. Herein, upon these theoretical insights, we developed spontaneous liquid‐phase reactions and self‐assembling process to in situ and massively precipitate highly exposed ZnO(100) and ZnO(002) onto Zn foils, denoted as ZnO(100)@Zn and ZnO(002)@Zn, respectively. Experimental results prove that the in situ formed ZnO(002) protective layer with thermodynamic inertia and kinetic zincophilicity effectively optimizes and stabilizes Zn anode during long‐term cycling process, while the ZnO(100) promotes rapid dendrite growth and induces side reactions, finally failing battery property. Consequently, the ZnO(002)@Zn cell enables stable cycling performance for up to 4000 h at 0.5 mA cm^−2^/0.5 mAh cm^−2^ and 500 h even at a larger DOD of 85.6%. Moreover, the ZnO(002)@Zn||V_2_O_5_ coin cell delivers a high specific capacity of 113 mAh g^−1^ over 1000 cycles at 1 A g^−1^ and the pouch cell attains a notable discharge capacity of 80.6 mAh with an ultrahigh V_2_O_5_ loading mass of 20.2 mg cm^−2^.

## 2 Results and Discussion

To identify the most suitable ZnO lattice plane for Zn deposition, the investigation into the stable thermodynamics and electrochemical kinetics of various substrates was performed via theoretical simulations. The interface between the Zn(002) and ZnO(002) planes shows the lowest energy of −0.51 eV (Figure 1b), indicating a robust interface interaction. The heightened chemical affinity and selectivity of Zn(002) for the ZnO(002) plane can greatly enhance and guide the homogeneous Zn deposition laterally along the ZnO(002) substrate.^[^ ^18^ ^]^ In contrast, the ZnO(101) plane displays a relatively lower Zn(002) affinity with an interface energy of −0.29 eV. Note that the ZnO(100) plane manifests a stronger chemical affinity with Zn(101) over with Zn(002), pointing toward a predilection for disordered Zn deposition and finally dendrite growth. The lowest Zn adsorption energy on the ZnO(002) plane provides further evidence that Zn^2+^ ions preferentially deposit on such a substrate (Figure S3, Supporting Information). Moreover, the ZnO(002) plane exhibits a more positive ∆G_H*_ (0.03 eV) compared to other substrates of Zn(101), Zn(002), ZnO(100), and ZnO(101) planes (Figure 1c), underscoring its excellent chemical inertness of HER among all candidates. Conversely, both ZnO(100) and ZnO(101) planes exhibit lower ∆G_H*_ values (−2.0 and −3.1 eV, respectively), suggesting easier formation of hydrogen on these substrates.^[^ ^19^ ^]^ The Bader charges (Figure S4, Supporting Information) and the differences in interfacial charge density (Figure S5, Supporting Information) were computed to study the charge transfer from the adsorptive atoms to the substrates. A more delocalized electronic distribution and higher Bader charge between Zn atom and ZnO(002) indicate that the ZnO(002) can effectively accelerate Zn^2+^→Zn^0^ reaction kinetics, also inhibit the aggregation of Zn deposition. Additionally, for the adsorption of H atoms, the ZnO(002) plane delivers a much lower Bader charge of 0.43 e compared to Zn(002) (0.96 e), Zn(101) (0.86 e), ZnO(100) (0.65 e), and ZnO(101) (0.59 e), demonstrating the reduced likelihood of hydrogen evolution on ZnO(002). Therefore, it can be inferred that the semi‐coherent ZnO(002) plane with Zn(002) can expedite uniform Zn deposition, mitigate Zn dendrite formation, and suppress HER (Figure 1d). Despite the good coherent interface of ZnO(101) with Zn(002), its weak thermodynamic stability and electrochemical kinetics of the Zn deposition are unfortunate to establish stable Zn anode in comparison with ZnO(002) plane. Moreover, owing to the zincophoibcity, low ∆G_H*,_ and high Zn(101) affinity, the ZnO(100) plane would lead to severe dendrite formation and side reactions.

**Figure 2:**
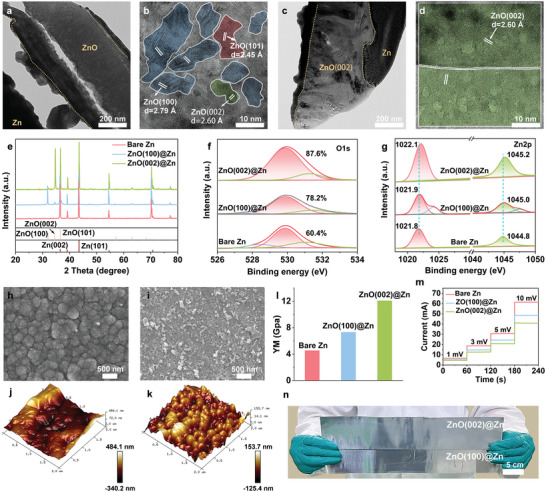
TEM and high‐resolution TEM images of a,b) ZnO(100)@Zn and c,d) ZnO(002)@Zn. e) XRD patterns of bare Zn, ZnO(100)@Zn and ZnO(002)@Zn. XPS spectra of f) O 1s and g) Zn 2p. SEM images of h) ZnO(100)@Zn and i) ZnO(002)@Zn anodes. AFM images of j) ZnO(100)@Zn and k) ZnO(002)@Zn. l) The detected Young's modulus on the bare Zn, ZnO(100)@Zn and ZnO(002)@Zn. m) Current responses to the potentials of 1, 3, 5, and 10 mV of bare Zn, ZnO(100)@Zn and ZnO(002)@Zn. n) The digital photos of the prepared ZnO(100)@Zn and ZnO(002)@Zn.

Based on these discoveries and deductions, highly oriented ZnO(002) facets with high zincophilicity were well‐prepared onto the Zn anode through spontaneous liquid‐phase redox reactions and self‐assembling process. In comparison, ZnO(100)‐protected Zn anode was also prepared. Transmission electron microscopy (TEM) and high‐resolution TEM images unveil that the interphase of ZnO(100)@Zn consists predominantly of the ZnO(100) lattice, along with a small presence of ZnO(101) and ZnO(002) lattices (**Figure** **2**a,b), whereas a highly crystalline ZnO(002) interphase can be observed on ZnO(002)@Zn (Figure 2c,d). X‐ray diffraction (XRD) patterns unambiguously demonstrate intense signal peaks at 31.8° for the ZnO(100) plane on ZnO(100)@Zn and at 34.7° for the ZnO(002) plane on ZnO(002)@Zn anodes (P63mc, JCPDS: 75–1526), alongside typical characteristic peaks of Zn metals, with almost negligible additional diffraction peaks of ZnO (Figure 2e). Moreover, the ratio (I_(002)/(101)_) values of Zn(002) to Zn(101) of ZnO(100)@Zn and ZnO(002)@Zn anodes resemble the value of bare Zn anode (Figure S6, Supporting Information). These confirm the successful fabrication of ZnO(100) and ZnO(002) on the Zn surface. Surface chemical composition of anodes was analyzed by X‐ray photoelectron spectroscopy (XPS).^[^ ^20^ ^]^ In the O 1s spectra shown in Figure 2f, ZnO(002)@Zn shows a significantly higher proportion (87.6%) of lattice oxygen species at 529.9 eV than bare Zn (60.4%) and ZnO(100)@Zn (78.2%), rather than chemisorbed oxygen on the surface.^[^ ^21^ ^]^ Meanwhile, a notable red shift of Zn 2p binding energy is evident in ZnO(002)@Zn (Figure 2g) relative to bare Zn, attributable to the strong Zn─O polar bonds at the ZnO─Zn interface.^[^ ^22^ ^]^ The large electronegativity of oxygen atom facilitates substantial charge transfer and redistribution at the ZnO─Zn interface, consistent with the theoretical calculations and conducive to expediting the migration and deposition of Zn^2+^.^[^ ^23^ ^]^

The surface micromorphology of the anodes was observed by scanning electron microscopy (SEM). Differing from the smooth and featureless surface of the bare Zn anode (Figure S7, Supporting Information), the ZnO(100)@Zn is adorned with well‐arranged ZnO nanoparticles with the average thickness of 3.7 µm (Figure 2h and Figure S8a, (Supporting Information), while the ZnO(002)@Zn surface presents a 3D stacking of disordered and irregular nanosheet morphology with the average thickness of 2.4 µm (Figure 2i; Figure S8b, Supporting Information). Atomic force microscopy (AFM) images depict that the ZnO(100)@Zn shows a notably increased surface roughness with an altitude intercept of ≈600 nm (Figure 2j; Figure S10a, Supporting Information), compared to the bare Zn (Figure S9, Supporting Information). In contrast, the ZnO(002)@Zn maintains a relatively mild surface roughness of 130 nm (Figure 2k; Figure S10b, Supporting Information). The hydrophilicity of the ZnO(100) and ZnO(002) interphases was assessed through water contact angle experiments (Figure S11, Supporting Information). The contact angle on ZnO(002)@Zn is measured as 48.3°, significantly lower than on the bare Zn (94.3°) and ZnO(100)@Zn (68.1°). This difference is attributed to the 3D stacking of ZnO(002) facets with abundant lattice oxygen, promoting the formation of strong hydrogen bonds with water molecules.^[^ ^3^, ^24^ ^]^ It is widely acknowledged that a robust and resilient SEI layer effectively withstands the cyclical volume fluctuations during the repeated Zn deposition and dissolution processes and prevents dendrite growth in regions with weak mechanical strength.^[^ ^25^ ^]^ Nanoindentation measurements of the anodes were carried out, and the force‐displacement curves (Figure S12, Supporting Information) demonstrate that the ZnO(002)@Zn anode exhibits a more reversible shape compared to the bare Zn and ZnO(100)@Zn anodes, indicating an elastic deformation feature with high reversibility, as opposed to the elastic‐plastic deformation observed in the bare Zn and ZnO(100)@Zn.^[^ ^26^ ^]^ Importantly, a higher and more uniform Young's modulus of 12.1 GPa can be obtained on ZnO(002)@Zn, surpassing that on bare Zn (4.5 Gpa) and ZnO(100)@Zn (7.3 GPa) (Figure 2l; Figure S13, Supporting Information). In addition, the insulating ZnO(002) layer exhibits an electronic resistance of 50.8 Ω cm^−2^ (Figure 2m), which is higher than bare Zn (0.8 Ω cm^−2^) and ZnO(100) layer (27.8 Ω cm^−2^). This higher resistance enables the ZnO(002) insulation layer to isolate the conductive Zn metal from aqueous electrolyte and prevent water‐induced side reactions. Moreover, the facile preparation process allows for the easy fabrication of the ZnO(002)@Zn and ZnO(100)@Zn anodes to 320 cm^2^ (Figure 2n).

**Figure 3:**
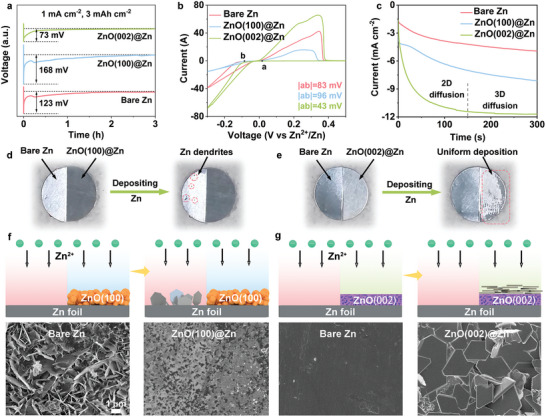
a) The nucleation overpotential of Zn on different anodes. b) CV curves of Zn||Cu cells. c) CA curves of Zn||Zn cells. The digital photographs of Zn depositing on the electrodes of d) bare Zn‐ZnO(100)@Zn and e) bare Zn‐ZnO(002)@Zn. SEM images after Zn depositing on f) bare Zn‐ZnO(100)@Zn and g) bare Zn‐ZnO(002)@Zn at 1 mA cm^−2^ for 5 min.

To validate the aforementioned hypotheses and assess the efficacy of different anode designs, the nucleation overpotentials of Zn in various Zn||Zn cells were first measured. As shown in **Figure** **3**a, the Zn nucleation overpotential of the ZnO(002)@Zn anode manifests notably lower at 73 mV, while the ZnO(100)@Zn counterpart exhibits a larger overpotential (168 mV) than bare Zn (123 mV). This observation underscores the efficacy of the ZnO(002) protective layer in mitigating the Zn nucleation barrier on anode, thereby facilitating fast ion deposition kinetics, while the ZnO(100)@Zn configuration poses a substantial impediment to Zn nucleation. Cyclic voltammetry (CV) profiles of Zn||Cu asymmetric cells employing different anodes also evidence these findings, which the ZnO(002)@Zn electrode possesses diminished nucleation overpotential and heightened deposition kinetics compared to its sluggish counterpart, ZnO(100)@Zn (Figure 3b).^[^ ^27^ ^]^ Chronoamperometry (CA) measurements shed further light on the nucleation and deposition dynamics of the anodes (Figure 3c). Application of a potential of −150 mV to Zn||Zn cells reveals that the ZnO(002)@Zn anode achieves prompt stabilization of the current density following 150 s of 2D longitudinal diffusion, indicative of a 3D diffusion process governing the movement of Zn^2+^ on the surface of ZnO(002)@Zn, thus impeding the agglomeration of Zn and the formation of protrusions. Conversely, the bare Zn adheres to a typical 2D ion diffusion model, characterized by a continuous decline in current density, and ZnO(100)@Zn exhibits an even more pronounced manifestation of this behavior. To visually elucidate these contrasting behaviors between ZnO(100)@Zn and ZnO(002)@Zn, an electrode comprising half bare Zn and half ZnO(100)@Zn (or ZnO(002)@Zn) was fabricated, followed by the deposition of Zn at a current density of 1 mA cm^−2^ for 5 min. As shown in Figure 3d, after deposition, the bare Zn segment shows numerous protrusions of deposited Zn, while the ZnO(100)@Zn section remains clean and devoid of any Zn accumulation, implying that the ZnO(100) protective layer diminishes chemical affinity toward Zn^2+^ ions and retards deposition kinetics. As for the electrode featuring the bare Zn and ZnO(002)@Zn, Zn^2+^ ions selectively nucleate and deposit solely on the ZnO(002)@Zn portion, bypassing the bare Zn (Figure 3e). SEM images corroborate these observations that compared to the bare Zn, the ZnO(100) layer exhibits a tendency to restrain and even repel Zn deposition to some extent (Figure 3f; Figure S14, Supporting Information), while the ZnO(002) with its heightened zincophilicity has a preferential affinity for Zn deposition and can facilitate uniform deposition along Zn(002) plane (Figure 3g).

**Figure 4:**
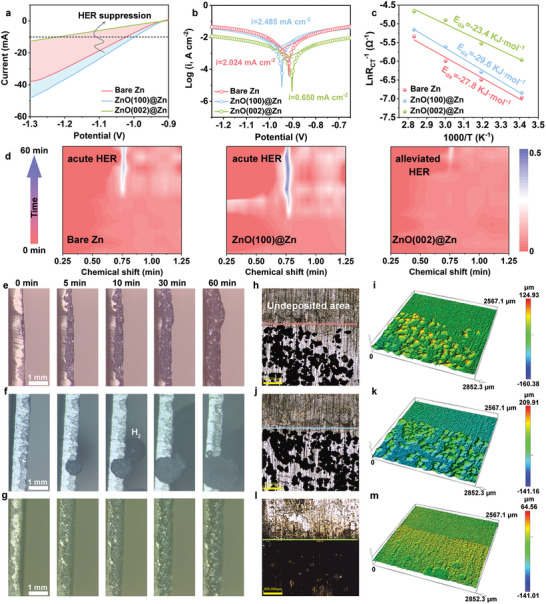
a) LSV curves, b) Tafel plots, and c) desolvation energy of Zn^2+^ ions on different anodes. d) In situ GC results during the Zn plating process at 10 mA cm^−2^/10 mAh cm^−2^. In situ optical microscope images of (e) bare Zn, (f) ZnO(100)@Zn, and (g) ZnO(002)@Zn. CLMS images of Zn depositing on (h, i) bare Zn, (j, k) ZnO(100)@Zn, and (l, m) ZnO(002)@Zn after in situ optical microscope experiments.

The HER performances of different anodes were detected through linear sweep voltammetry (LSV) illustrated in **Figure** **4**a. The ZnO(002)@Zn always produces a smaller current density compared to the bare Zn and ZnO(100)@Zn, indicating an attenuated HER kinetics due to the protective ZnO(002) layer, well consistent with the theoretical predictions of ∆G_H*_. Meanwhile, the ZnO(002)@Zn also shows a lower corrosion resistance (0.650 mA cm^−2^) than the bare Zn (2.024 mA cm^−2^) and ZnO(100)@Zn (2.485 mA cm^−2^), confirming the superior anti‐corrosion performance of ZnO(002)@Zn (Figure 4b). The subdued HER activity arises from the insulating nature of the ZnO(002) layer with rich lattice oxygen, effectively sequestering water molecules and hindering their electron uptake for decomposition.^[^ ^3^ ^]^ Moreover, the ZnO(002)@Zn anode demonstrates lower charge transfer resistance (R_CT_) (Figure S15, Supporting Information) and desolvation activation energy (E_da_) of hydrated Zn^2+^ (23.4 kJ mol^−1^) (Figure 4c) relative to the bare Zn (27.8 kJ mol^−1^) and ZnO(100)@Zn (29.6 kJ mol^−1^), signifying improved reaction kinetics by ZnO(002) protection. In situ gas chromatography (GC) was employed to directly ascertain the HER activity of different anodes during the Zn plating process at 10 mA cm^−2^/10 mAh cm^−2^. Notably, the bare Zn anode exhibits a prominent H_2_ peak intensity at the chemical shift of 0.75 min, and the ZnO(100)@Zn anode dramatically enlarges the peak intensity, meaning their reduced thermodynamic stability and HER resistance. On the contrary, a minimal H_2_ signal is monitored on the ZnO(002)@Zn anode, indicating its superior performance in mitigating HER activity (Figure 4d).

The dendrite growth process as well the HER of the anodes were scrutinized via in situ optical microscope at the current density of 10 mA cm^−2^. For the bare Zn (Figure 4e), dendritic formations of Zn commence at 10 min, gradually deteriorating during the plating process. Owing to the inferior zincophilicity and thermodynamic stability of the ZnO(100)@Zn anode, the pronounced dendritic growth along the edges and conspicuous H_2_ bubble formation are observed (Figure 4f), exacerbating the uneven distribution of charge density and Zn^2+^ concentration and speeding up dendrite growth and side reactions. As expected, the ZnO(002)@Zn anode shows a uniform and glossy Zn deposition layer without dendrites and bubbles after 60 min, indicating effective deposition and suppression of side reactions. These observations were further affirmed by confocal laser microscopy (CLMS). As shown in Figure 4h–k, the bare Zn and ZnO(100)@Zn anodes present an uneven and patchy surface with high protrusions, attributed to the severe dendrite formation and growth. As a comparison, the ZnO(002)@Zn anode yields a uniform and dense Zn deposition, with distinct demarcation between deposited and undeposited regions (Figure 4i,m). The Zn deposition behavior of the anodes was also characterized by SEM images. Upon depositing at different capacity densities (1 and 5 mAh cm^−2^), mountainous dendrites and coarse Zn morphologies are formed on the bare Zn and ZnO(100)@Zn surfaces, while a homogeneous Zn surface with a characteristic Zn(002) texture is evident for the ZnO(002)@Zn (Figures S16–S18, Supporting Information). Corresponding XRD patterns confirm the predominant deposition behavior along the Zn(002) facilitated by ZnO(002) interphase (Figure S19, Supporting Information). Such results elaborate that the bare Zn and ZnO(100)@Zn anodes are prone to dendrite growth and side reactions, ultimately compromising the performance of Zn batteries, but the ZnO(002) protection layer featured with high zincophilicity, uniform deposition, and low HER kinetics, ensure the stability and longevity of Zn anodes (Figure 1d).

**Figure 5:**
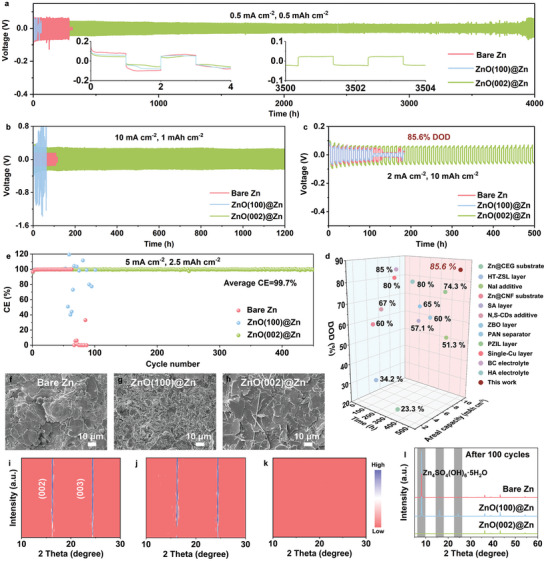
Cycling performances of Zn||Zn cells at (a) 0.5 mA cm^−2^/0.5 mAh cm^−2^, b) 10 mA cm^−2^/1 mAh cm^−2^, and c) 2 mA cm^−2^/10 mAh cm^−2^. e) CE of Zn||Cu cells at 5 mA cm^−2^/2.5 mAh cm^−2^. d) Comparison of cycling performance of Zn||Zn cells. SEM images of f) bare Zn, g) ZnO(100)@Zn, and h) ZnO(002)@Zn after 100 cycles. In situ XRD patterns of i) bare Zn, j) ZnO(100)@Zn, and k) ZnO(002)@Zn at the current density of 10 mA cm^−2^. l) XRD patterns after cycling.

To validate the benefits of the ZnO(002) layer on Zn anodes, the galvanostatic cycling performance of Zn||Zn cells was characterized at various current densities and areal capacities. The bare Zn anodes show a sudden voltage collapse after cycling for 287 h at 0.5 mA cm^−2^/0.5 mAh cm^−2^ (**Figure** **5**a). As expected, the ZnO(100)@Zn anode experiences a rapid short circuit only after cycling for 26 h, significantly worse than bare Zn anodes. However, the ZnO(002)@Zn anode exhibits an extended cycling lifespan up to 4000 h, almost 14 and 154 times longer than the bare Zn and ZnO(100)@Zn anodes, respectively. Even under altering current densities from 0.5 to 10 mA cm^−2^ and then reverting to 0.5 mA cm^−2^, the ZnO(002)@Zn anodes also exhibit low and reversible voltage hysteresis (Figure S20, Supporting Information). Moreover, at higher current densities of 5 mA cm^−2^/2.5 mAh cm^−2^ and 10 mA cm^−2^/1 mAh cm^−2^, the ZnO(002)@Zn can run steadily for over 1 000 h with the cumulative deposition capacity (CDC) of 1250 mAh cm^−2^ (Figure S21, Supporting Information) and 1,200 h with the CDC of 6000 mAh cm^−2^ (Figure 5b), respectively. Even at ultrahigh current densities of 20 mA cm^−2^/5 mAh cm^−2^ and 40 mA cm^−2^/1 mAh cm^−2^, it still exhibits better cycling stability than the bare Zn and ZnO(100)@Zn anodes (Figures S22 and S23, Supporting Information). When a high DOD of 85.6% with the current density of 2 mA cm^−2^/10 mAh cm^−2^ is employed, the ZnO(002)@Zn anodes maintain much better cycling performance of 500 h compared to the bare Zn (120 h) and ZnO(100)@Zn (105 h) anodes (Figure 5c). It is demonstrated that the constructed ZnO(002) protection layer has a satisfactory ability to prevent dendrite growth and stabilize Zn anode, even under ultrahigh cycling current and capacity. Remarkably, the cycling lifespan achieved by ZnO(002)@Zn anode, with the corresponding DOD, is also much higher than that of previously reported Zn||Zn cells based on various electrodes, electrolytes, and separators modification strategies (Figure 5d).^[^ ^1^, ^7^, ^28^ ^]^

To assess the impact of the ZnO(002) layer on Zn plating/stripping reversibility, the CE of the anodes was measured in Zn||Cu asymmetric cells. The ZnO(002)@Zn anode causes stable operations over 400 cycles with an average CE of 99.2% at 0.5 mA cm^−2^/0.5 mAh cm^−2^ (Figure S24, Supporting Information) and 450 cycles with an average CE of 99.7% at 5 mA cm^−2^/2.5 mAh cm^−2^ (Figure 5e), showcasing splendid durability and reversibility. By contrast, the cycling performance of the bare Zn and ZnO(100)@Zn anodes abruptly fails after only a few cycles. SEM images reveal that after cycling, serious protrusions and rough surface are obvious on the bare Zn (Figure 5f) and ZnO(100)@Zn (Figure 5g), while the ZnO(002)@Zn anode presents a dendrite‐free morphology with favorable deposition manner of Zn(002) planes (Figure 5h). Additionally, the ZnO(002) protective layer maintains similar thickness with before cycling (Figure S25, Supporting Information), indicating its good stability and adhesion to Zn substrate. In situ, XRD patterns on the electrodes were used to monitor the by‐product generation during the Zn plating process at a current density of 10 mA cm^−2^ for 60 min. The bare Zn and ZnO(100)@Zn rise strong XRD peaks at 16.2° and 24.4° (Figure 5i,j), corresponding to (002) and (003) planes of Zn_4_SO_4_(OH)_6_∙5H_2_O (ZSH) (JCPDS, PDF#39‐0688) by‐product,^[^ ^29^ ^]^ respectively. However, there are no any characteristic peaks of ZSH detected on the ZnO(002)@Zn throughout the whole plating process (Figure 5k). Moreover, after undergoing 100 cycles of Zn plating/stripping, the ZnO(100)@Zn displays a more dramatic XRD signal of ZSH than the bare Zn, and the ZnO(002)@Zn still keeps a clean surface without any impurities (Figure 5l). Additionally, XPS spectra of S 2p also show a similar trend as shown in Figure S26 (Supporting Information). Nyquist plots of Zn||Zn cells at various cycles demonstrate that the formation and accumulation of by‐products on the Zn surface can critically and detrimentally elevate the R_CT_ of cells and impede charge/ion transfer (Figure S27, Supporting Information).^[^ ^30^ ^]^ Consequently, it can be inferred that the (002) lattice plane of ZnO with high zincophilicity efficiently enhances Zn^2+^ kinetics, modulates Zn deposition behavior and suppresses various side reactions, thus achieving ultra‐stable Zn anodes, while the (100) plane of ZnO is easy to trigger dendrite growth, HER and by‐product generation, leading to rapid battery failure.

**Figure 6:**
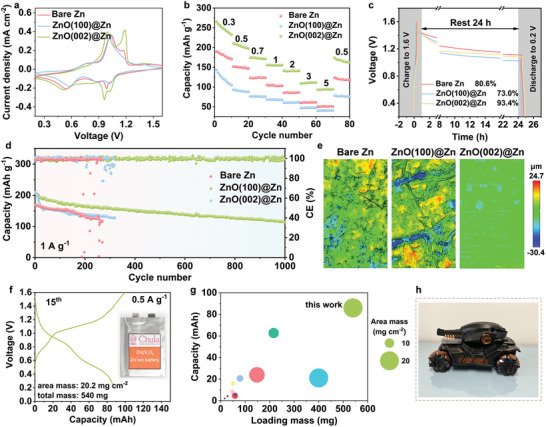
a) CV curves, b) rate performance, and c) resting measurements of Zn||V_2_O_5_ full cells. d) Long‐term cycling performance at 1 A g^−1^. e) CLMS 2D images of Zn anodes after cycling. f) Voltage profile of ZnO(002)@Zn||V_2_O_5_ pouch cells. g) Comparison of discharge capacities of pouch cells. h) Pouch cells running a toy car.

ZnO(002)@Zn anodes were meticulously assembled in conjunction with traditional V_2_O_5_ cathode materials to unlock their latent potential for real‐world deployment. The XRD pattern (Figure S28, Supporting Information) and SEM image (Figure S29, Supporting Information) distinctly delineate the successful preparation of V_2_O_5_. The CV curve of the ZnO(002)@Zn||V_2_O_5_ cell shows an augmented redox current in comparison to the bare Zn and ZnO(100)@Zn, attributed to its active Zn^2+^ reaction kinetics (**Figure** **6**a). Subsequently, the rate performance of Zn||V_2_O_5_ cells underwent rigorous testing across various current densities ranging from 0.3 to 5 A g^−1^. As illustrated in Figure 6b, the ZnO(002)@Zn full cells manifest commendable and reversible discharge capacity overall current densities, superior to the bare Zn and ZnO(100)@Zn. Nyquist plots of the ZnO(002)@Zn||V_2_O_5_ full cells before and after rate performances portray decreased battery resistances than the bare Zn and ZnO(100)@Zn (Figure S30, Supporting Information), which discloses an excellent ion transfer and proficiently mitigated side reactions enabled by ZnO(002)@Zn. Benefiting from this, a remarkable capacity retention of 93.4% can be achieved by the ZnO(002)@Zn after a resting period of 24 h, much greater than the bare Zn (80.6%) and ZnO(100)@Zn (73.0%) (Figure 6c). Long‐term cycling measurement was also carried out for the ZnO(002)@Zn anode at the current density of 1 A g^−1^. The ZnO(002)@Zn cells exhibit stable operation with a high discharge capacity of 113 mAh g^−1^ after 1000 cycles, while the bare Zn and ZnO(100)@Zn full cells succumb to device failure after ≈300 cycles (Figure 6d). To gain deeper insights into the performance enhancement of full cells based on ZnO(002)@Zn, 2D CLMS images of the anodes after 200 cycles were conducted and are portrayed in Figure 6e. The cycled ZnO(002)@Zn anode displays an even and seamless morphology, while the bare Zn and ZnO(100)@Zn anodes have numerous small ravines and protrusions scattered across the surface, indicating that the ZnO(002) protective layer bestows homogeneous Zn deposition/dissolution onto the Zn anode. These sights again appear in the SEM images and XRD patterns (Figures S31 and S32, Supporting Information). We also assembled a ZnO(002)@Zn||V_2_O_5_ pouch cell (5.6 × 4.8 cm^2^) with an industry‐standard average loading mass of 20.2 mg cm^−2^ and a total mass of 540 mg. The prepared pouch cell has good cycling stability at the current density of 0.05 A g^−1^ (Figure S33, Supporting Information) and its discharge capacity reaches 86.0 mAh (Figure 6f), which surpasses most of the reported Zn‐ion pouch cells (Figure 6g).^[^ ^2^, ^4^, ^11^, ^31^ ^]^ A practical demonstration involving five pouch cells in series smoothly powering a toy car is taken in Figure 6h and Video S1 (Supporting Information), providing a tangible of the potential of ZnO(002)@Zn in the realm of AZIBs.

## 3 Conclusion

In summary, we have engineered and prepared two distinct types of Zn electrodes on a large scale, namely ZnO(100)@Zn and ZnO(002)@Zn, on the basis of thermodynamic and kinetic criteria, aiming to tackle the intricate challenges associated with Zn metal. The combined theoretical and experimental investigations demonstrate the superior performance of the ZnO(002)@Zn anode, characterized by remarkable attributes such as heightened zincophilicity, enhanced ion transfer kinetics, mitigated side reactions, and uniform Zn deposition along the Zn(002) plane. Conversely, the presence of the ZnO(100) protective layer exacerbates issues including dendrite formation, HER, and Zn surface heterogeneity. As a result, compared to the bare Zn and ZnO(100)@Zn, the ZnO(002)@Zn shows exceptional stability and longevity in cycling, even under substantial DOD up to 85.6%, and achieves a high average CE of 99.7%. Moreover, the assembled ZnO(002)@Zn full cell yields a reversible discharge capacity of 113 mAh g^−1^ at 1 A g^−1^ and its pouch cell with an enlarged mass loading of 20.2 mg cm^−2^ showcases an impressive capacity of 80.6 mAh, outperforming competing systems. This comprehensive study not only establishes a rational framework for the selection and design of SEIs, but also heralds a reliable pathway toward the realization of highly stable Zn anodes.

## 4 Experimental Section

### Preparation of ZnO(100)@Zn Anode

A solution containing 2 mL H_2_O_2_, 3 mL NaOH (2 m), and 55 mL DI water was transferred to an autoclave. Zn foil with a size of 4.5 cm × 10 cm was immersed in the resultant solution. Then, the autoclave was heated at 130 °C for 3 h. After cooling down, the prepared ZnO(100)@Zn anode was washed with DI water and dried in the oven.

### Preparation of ZnO(002)@Zn Anode

A solution of 10 mL phenol in 50 mL ethanol was transferred to an autoclave. Zn foil with a size of 4.5 cm × 10 cm was immersed in the above solution and then heated at 130 °C for 12 h. After that, the prepared ZnO(002)@Zn anode was washed with DI water and dried in the oven.

### Preparation of V_2_O_5_ Cathode Material

1 g of NH₄VO₃ powder was put into a crucible and annealed to 400 °C for 2 h. After cooling down, the resultant V_2_O_5_ was collected and fully ground.

### Material Characterization

All the samples were investigated by X‐ray diffraction (XRD, Rigaku D X‐ray diffractometer), X‐ray photoelectron spectroscopy (XPS, Thermo ESCALAB 250XI), scanning electron microscopy (SEM. Hitachi S4800), 3D laser microscope (Olympus LEXT OLS5000), atomic force microscopy (AFM, Bruker Dimension ICON) and transmission electron microscope (TEM, Talos F200X G2) to achieve the information of composition, structures, elements and morphologies. The in situ XRD was tested in an electrochemical cell at the current density of 10 mA cm^−2^ with an areal capacity of 5 mAh cm^−2^. The in situ GC spectra were monitored by GC 2014C gas chromatography with a TCD detector.

### Theoretical Calculations

The calculation of this work based on density functional theory (DFT)^[^ ^32^ ^]^ was performed using the Vienna ab initio simulation package (VASP).^[^ ^33^ ^]^ Used projector augmented wave (PAW)^[^ ^34^ ^]^ and the Perdew‐Burke‐Ernzerhof (PBE)^[^ ^35^ ^]^ of generalized gradient approximation (GGA)^[^ ^36^ ^]^ exchange‐correlation functional to calculate self‐consistency and total energy. The plane‐wave basis cutoff was set at 550 eV and Gamma‐centered 3 × 5 × 1 k‐points mesh was applied to all calculations. The convergence accuracies for energy and stress are 1 × 10^−5^ eV per atom and 0.02 eV Å^−1^, respectively.

### Electrochemical Measurement

The cathode electrodes of V_2_O_5_ were prepared by coating the slurry of V_2_O_5_ powder, conductive carbon, and PVDF (7:2:1) onto carbon paper. The average loading mass of V_2_O_5_ was ≈2 mg∙cm^−2^. The Zn||Zn symmetric, Zn||Cu asymmetric, and Zn||V_2_O_5_ full cells were assembled in CR2032‐type cells with 2 m ZnSO_4_ aqueous solution as the electrolyte and the Whatman Glass fiber (GF/D) as the separator. The Zn||V_2_O_5_ pouch cells were also prepared, in which the cathode electrode of V_2_O_5_ with the size of 4.8 cm × 5.6 cm had an average loading mass of ≈25 mg∙cm^−2^. The galvanostatic cycling performances of cells at various current densities and areal capacities were tested on NEWARE battery testing system. The chronoamperometry (CA), Tafel curve, cyclic voltammetry (CV), linear sweep voltammetry (LSV), and electrochemical impedance spectra (EIS) were measured at CHI 660E workstation. The electronic resistivities of bare Zn, ZnO(100) and ZnO(002) were calculated by the equation:^[^ ^37^ ^]^

(1) $$\rho =\frac{R\times S}{L}=\frac{U\times S}{I\times L}$$

where U is the applied voltage; S is the surface area of the electrode; I is average current increase; L is the thickness of bare Zn, ZnO(100), and ZnO(002).

### Statistical Analysis

The data presentation in this work conforms the mean ± SD for the measurements of Young's modulus, force‐displacement curves, thickness of protective layers, electronic resistivities, Coulombic efficiencies, and various electrochemical performances of symmetric, asymmetric, and full cells, where n = 10 for each sample. All statistical analysis and drawing were done by Excel.

## Conflict of Interest

The authors declare no conflict of interest.

## Supporting information



Supporting Information

Supplemental Video 1

## Data Availability

The data that support the findings of this study are available from the corresponding author upon reasonable request.
