# Supplementary material for: Highly Reversible Zn Anode Design Through Oriented ZnO(002) Facets
Source: Adv Mater. 2024 Oct 21;36(49):2408908. doi: 10.1002/adma.202408908 (PMC11619232; doi:10.1002/adma.202408908)
Supplement: Supplementary file 1 — Supporting Information [file ADMA-36-2408908-s002.docx]

**Highly Reversible Zn Anode Design Through Oriented ZnO(002) Facets**

Chengwu Yang^a#^, Pattaraporn Woottapanit^a#^, Sining Geng^b#^, Kittima Lolupiman^a^, Xinyu Zhang^b^*, Zhiyuan Zeng^c,d^, Guanjie He^e^*, Jiaqian Qin^a^*

^a^Center of Excellence in Responsive Wearable Materials, Metallurgy and Materials Science Research Institute, Chulalongkorn University, Bangkok 10330, Thailand

^b^State Key Laboratory of Metastable Materials Science and Technology, Yanshan University, Qinhuangdao 066004, P. R. China

^c^Department of Materials Science and Engineering and State Key Laboratory of Marine Pollution, City University of Hong Kong, 83 Tat Chee Avenue, Kowloon, Hong Kong 999077, China

^d^Shenzhen Research Institute, City University of Hong Kong, Shenzhen 518057, China.

^e^Christopher Ingold Laboratory, Department of Chemistry, University College London, London WC1H 0AJ, UK

^#^ These authors contributed equally to this work.

*Corresponding Author. E-mail: [xyzhang@ysu.edu.cn](mailto:xyzhang@ysu.edu.cn) (X. Zhang), [g.he@ucl.ac.uk](mailto:g.he@ucl.ac.uk) (G. He), jiaqian.q@chula.ac.th (J. Qin)


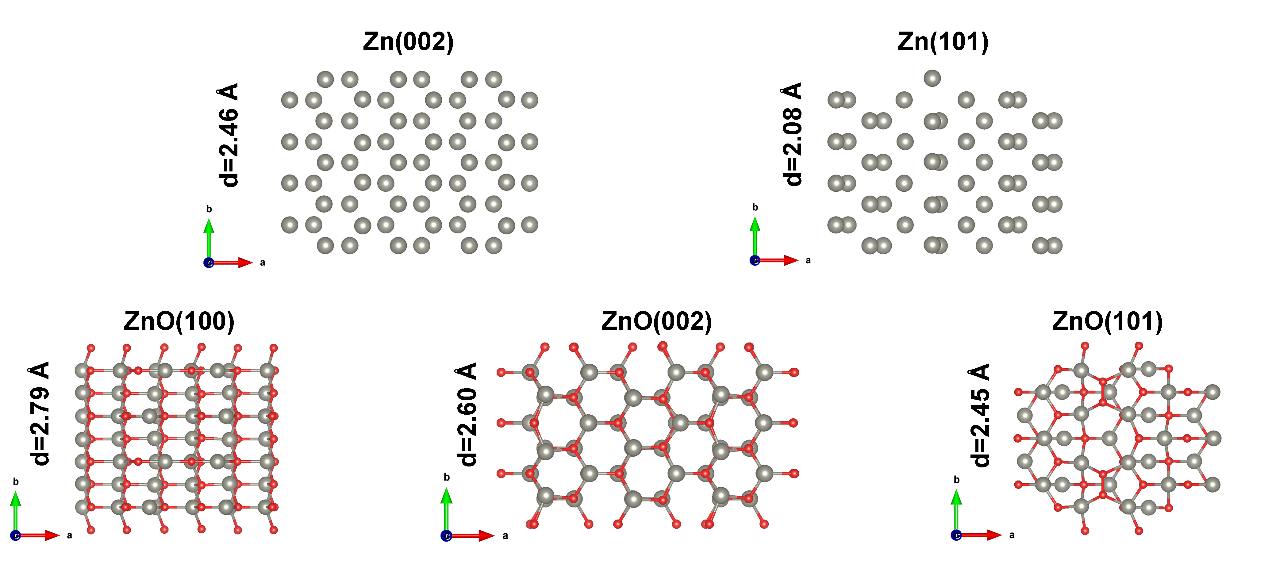


**Figure S1.** The stuctrure of Zn(002), Zn(101), ZnO(100), ZnO(002) and ZnO(101) lattice planes with the corresponding lattice distance.





**Figure S2.** The lattice mismatch of Zn(101) and Zn(002) on the substrates of ZnO(100), ZnO(002) and ZnO(101).

**

**

**Figure S3.** The calculated E_a_ of Zn atom on different substrates.

**

**

**Figure S4.** Bader charges of H and Zn atoms on the different substrates.


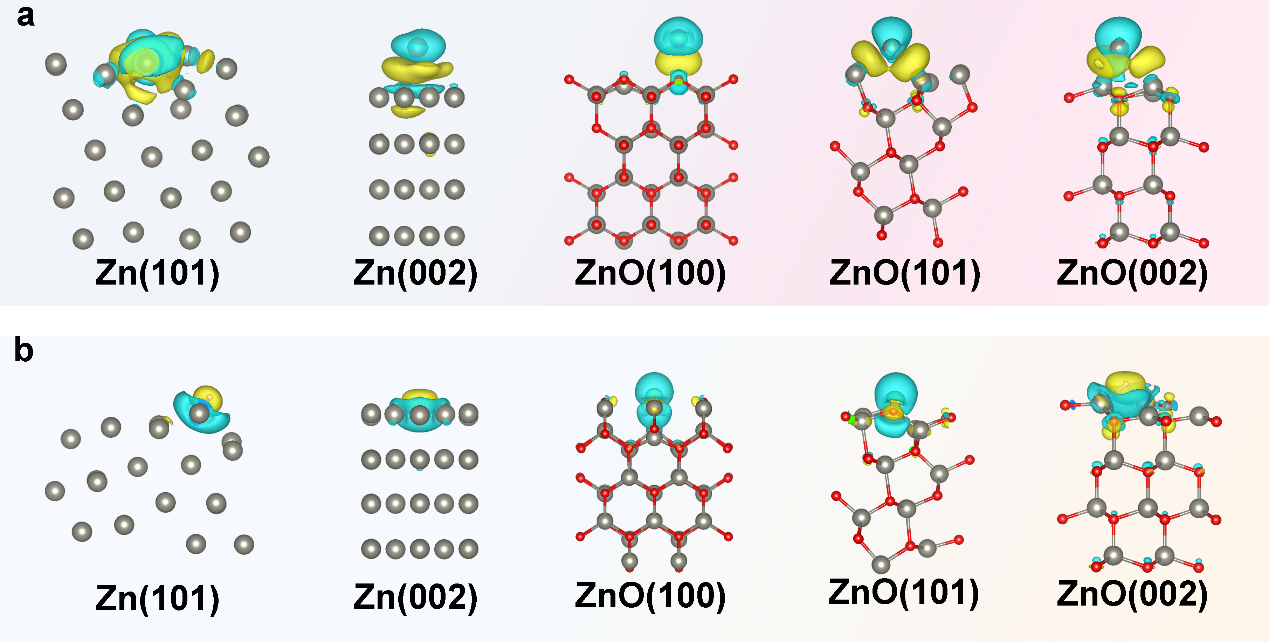


**Figure S5.** Difference of charge density distributions at the interface of (a) Zn atoms and (b) H atoms on the different substrates.





**Figure S6.** The I_(002)/(101)_ of bare Zn, ZnO(100)@Zn and ZnO(002)@Zn.


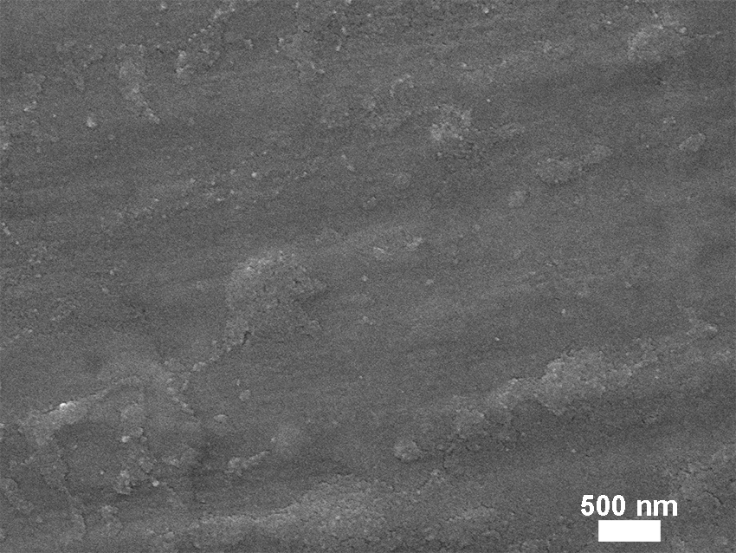


**Figure S7.** The SEM image of bare Zn anode.


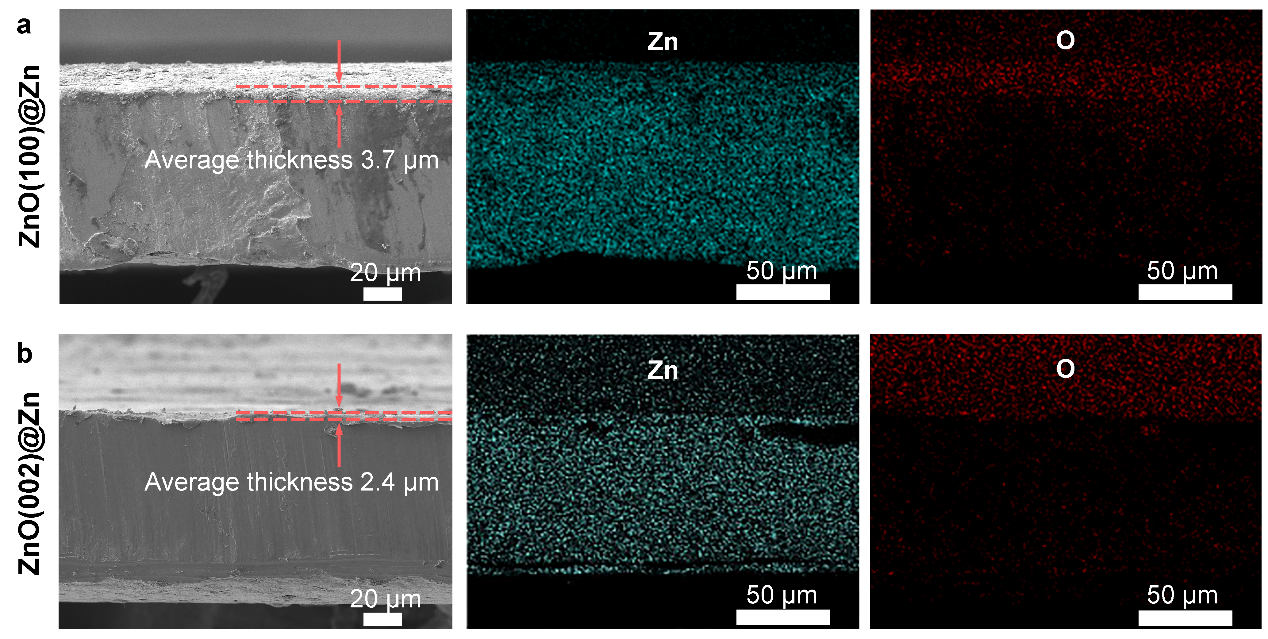


**Figure S8.** The SEM images of (a) ZnO(100)@Zn and (b) ZnO(002)@Zn at the cross-sectional view with the corresponding EDX element mapping.


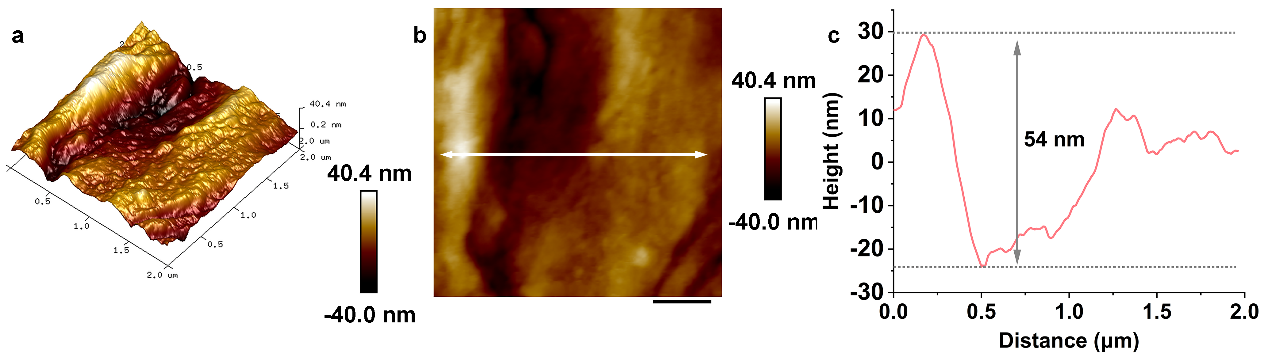


**Figure S9.** (a, b) AFM images of bare Zn anode and (c) the corresponding surface roughness.


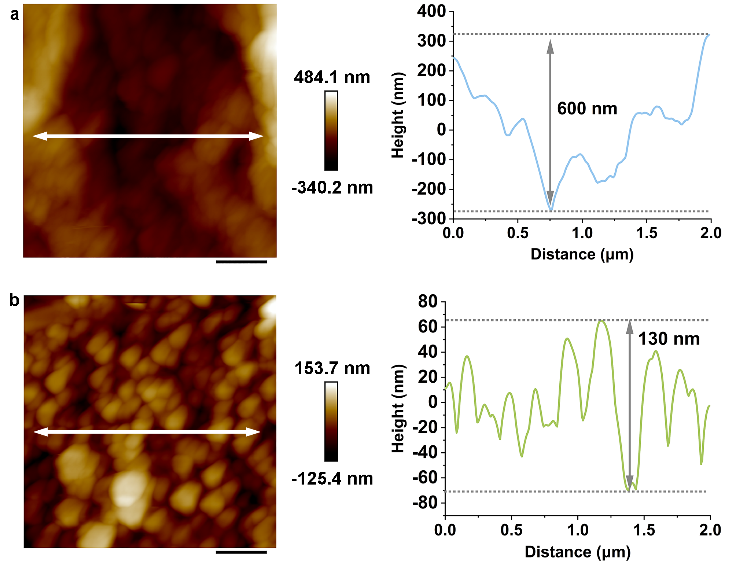


**Figure S10.** AFM images and the corresponding surface roughness of (a) ZnO(100)@Zn and (b) ZnO(002)@Zn.


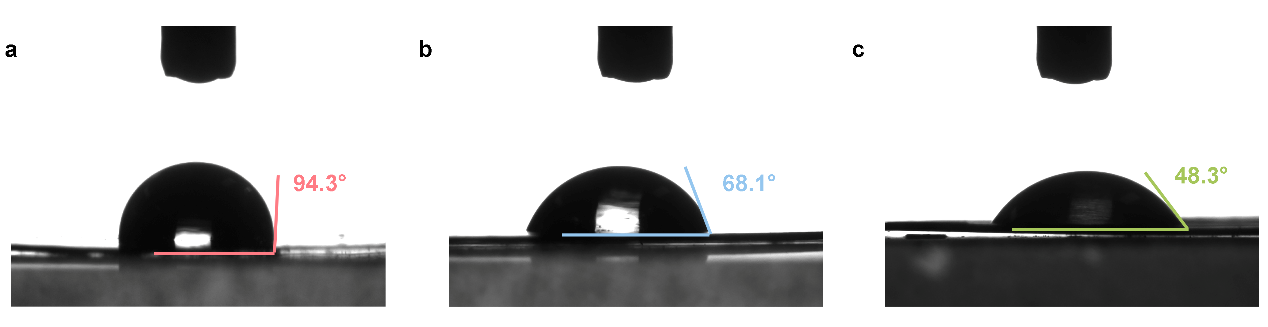


**Figure S11.** Water contact angle measurements of (a) bare Zn, (b) ZnO(100)@Zn and (c) ZnO(002)@Zn.





**Figure S12.** Force-displacement curves for nanoindentation measurements for bare Zn, ZnO(100)@Zn and ZnO(002)@Zn.


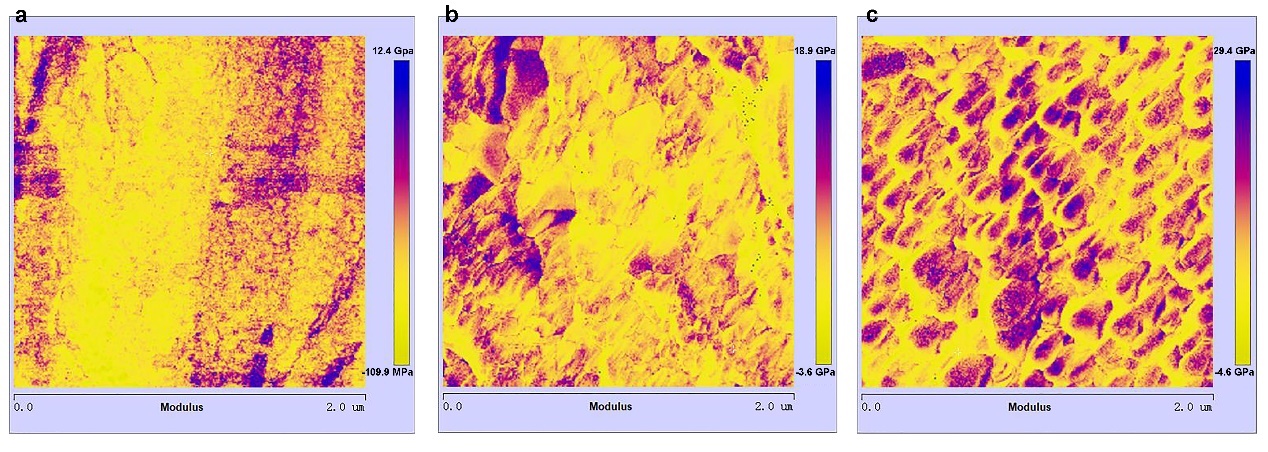


**Figure S13.** Surface map of Young’s modulus of (a) bare Zn, (b) ZnO(100)@Zn and (c) ZnO(002)@Zn.


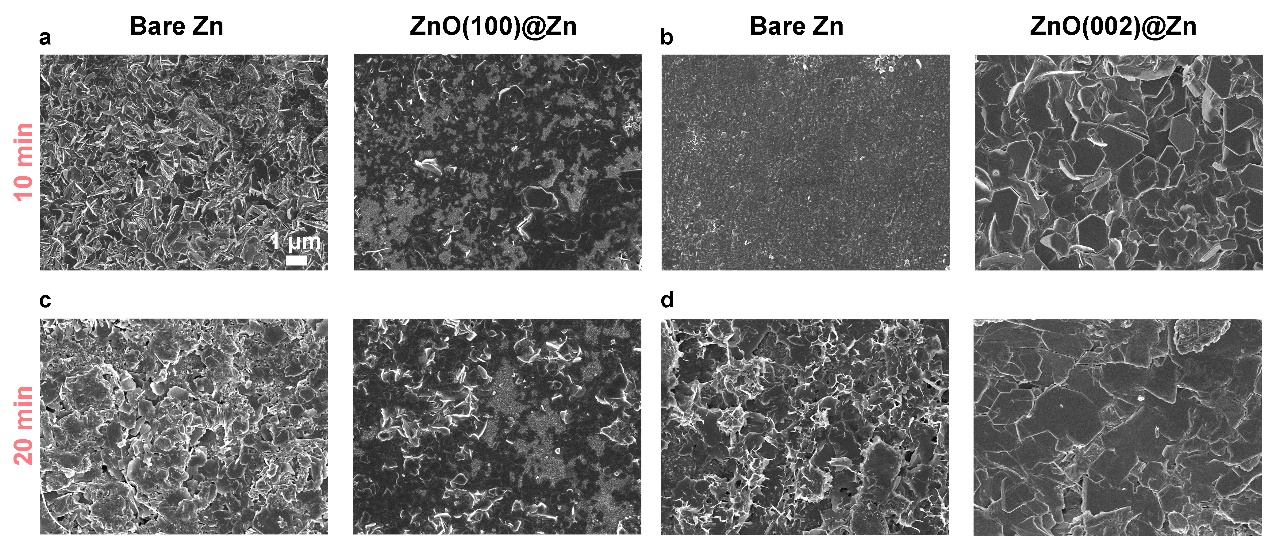


**Figure S14.** SEM images of after Zn deposition on (a, c) bare Zn-ZnO(100)@Zn and (b, d) bare Zn-ZnO(002)@Zn for 10 and 20 min. With the increasing time of Zn deposition, for the bare Zn-ZnO(002)@Zn, the bare Zn side obtains Zn^2+^ deposition, because the ZnO(002)@Zn side is covered by the Zn nanosheet during the Zn deposition process, resulting in the gradually increasing Zn nucleation barrier close to bare Zn. However, for the electrode of bare Zn-ZnO(100)@Zn, the ZnO(100)@Zn still does not have remarkable Zn harvest.


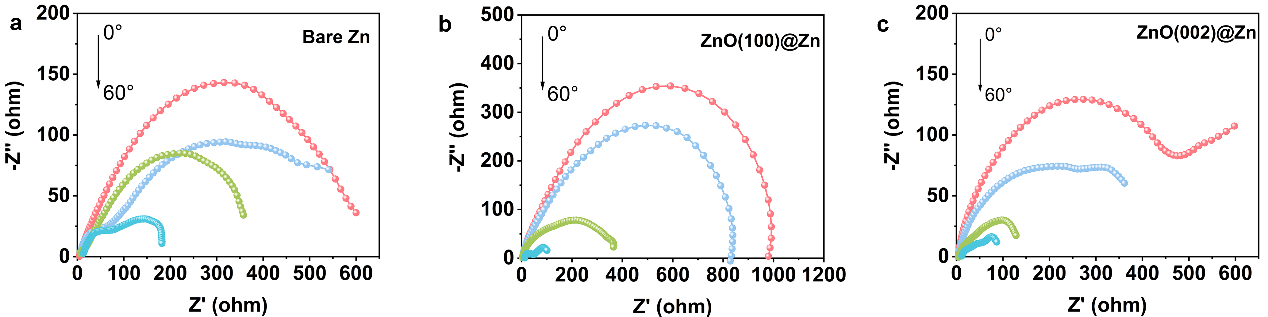


**Figure S15.** Nyquist plots of (a) bare Zn, (b) ZnO(100)@Zn and (c) ZnO(002)@Zn at different temperatures.


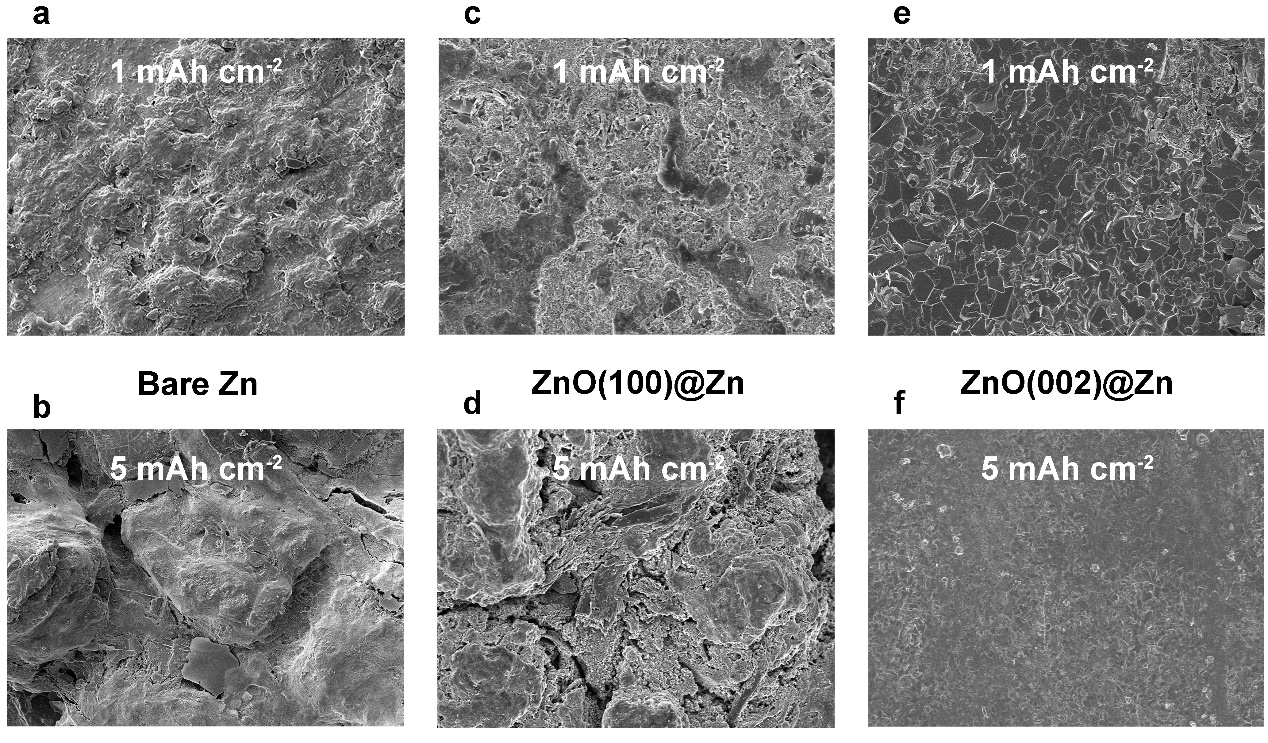


**Figure S16.** SEM images of (a, b) bare Zn and (c, d) ZnO(100)@Zn and (e, f) ZnO(002)@Zn after depositing Zn of 1 and 5 mAh cm^-2^.


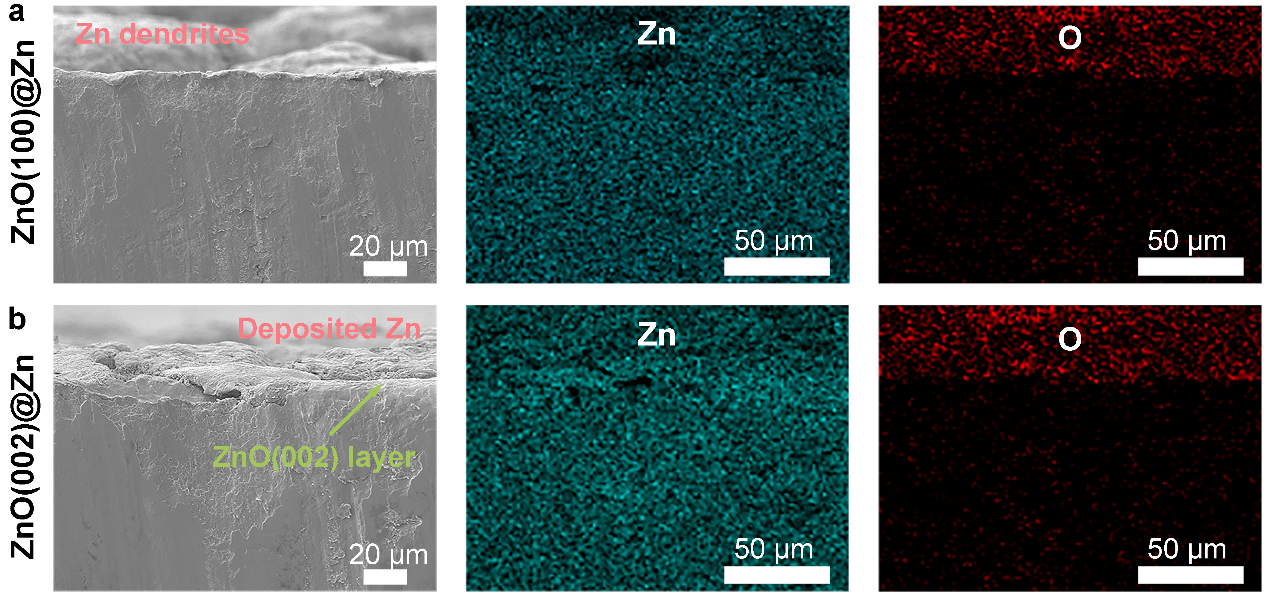


**Figure S17.** SEM images of (a) ZnO(100)@Zn and (b) ZnO(002)@Zn after depositing Zn of 5 mAh cm^-2^ at cross-sectional view with the corresponding EDX element mapping.


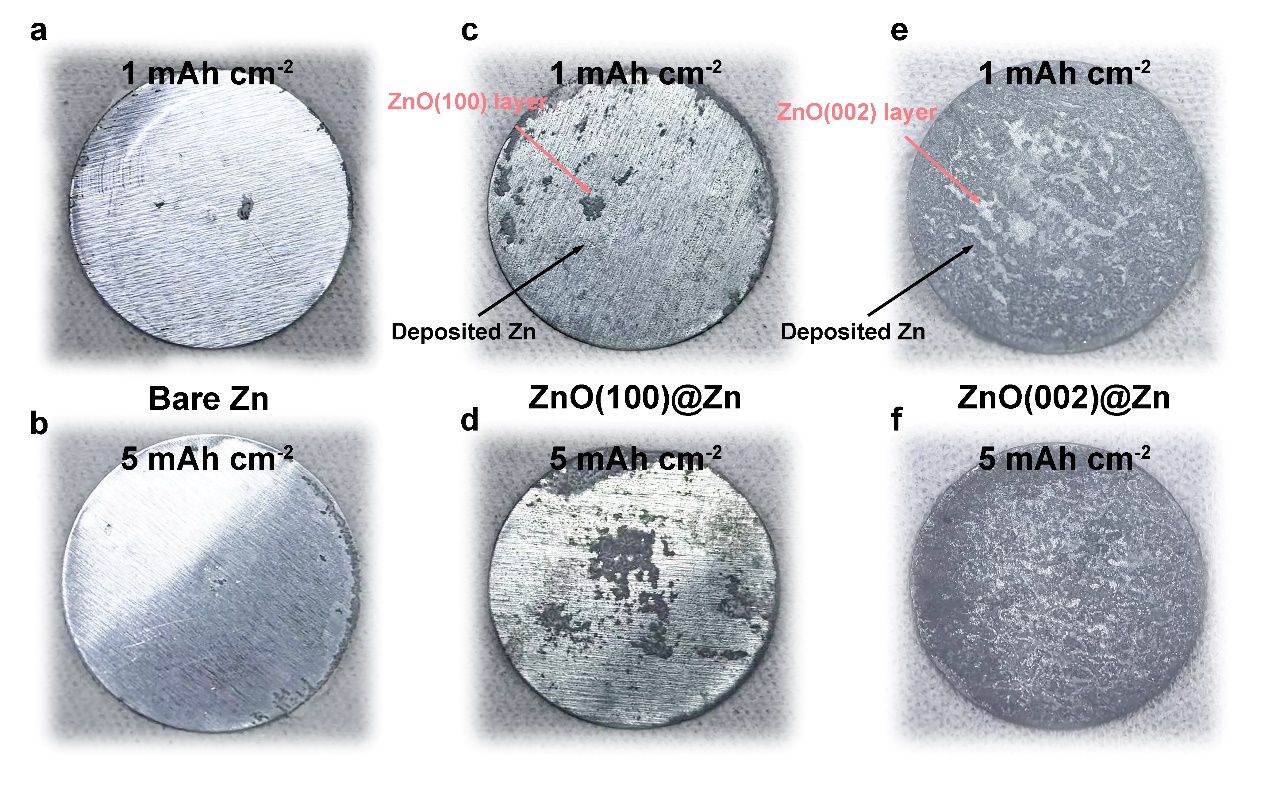


**Figure S18.** The digital photographs of (a, b) bare Zn and (c, d) ZnO(100)@Zn and (e, f) ZnO(002)@Zn after depositing Zn of 1 and 5 mAh cm^-2^.





**Figure S19.** XRD patterns of bare Zn, ZnO(100)@Zn and ZnO(002)@Zn after Zn deposition with the I_(002)_/I_(101)_.





**Figure S20.** The rate performance of bare Zn, ZnO(100)@Zn and ZnO(002)@Zn anodes at the current densities from 0.5, 1, 2, 3, 5, 10 mA cm^-2^ and backing to 0.5 mA cm^-2^..





**Figure S21.** The cycling performance of bare Zn, ZnO(100)@Zn and ZnO(002)@Zn anodes at the current density of 5 mA cm^-2^/2.5 mAh cm^-2^.





**Figure S22.** The cycling performance of bare Zn, ZnO(100)@Zn and ZnO(002)@Zn anodes at the current density of 20 mA cm^-2^/5 mAh cm^-2^.





**Figure S23.** The cycling performance of bare Zn, ZnO(100)@Zn and ZnO(002)@Zn anodes at the current density of 40 mA cm^-2^/1 mAh cm^-2^.





**Figure S24.** The CE of bare Zn, ZnO(100)@Zn and ZnO(002)@Zn anodes at 0.5 mA cm^-2^/0.5 mAh cm^-2^.


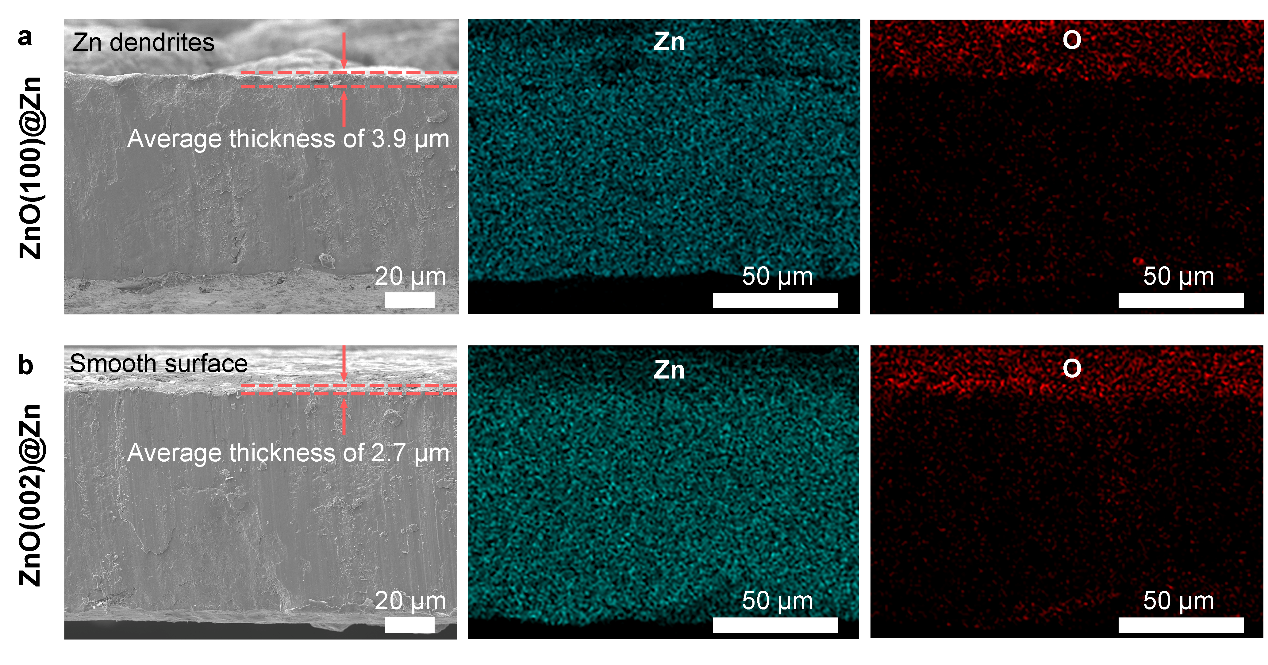


**Figure S25.** The SEM images of (a) ZnO(100)@Zn and (b) ZnO(002)@Zn at the cross-sectional view with the corresponding EDX element mapping after 50 cycles.





**Figure S26.** XPS S2p spectra of bare Zn, ZnO(100)@Zn and ZnO(002)@Zn after 100 cycles.


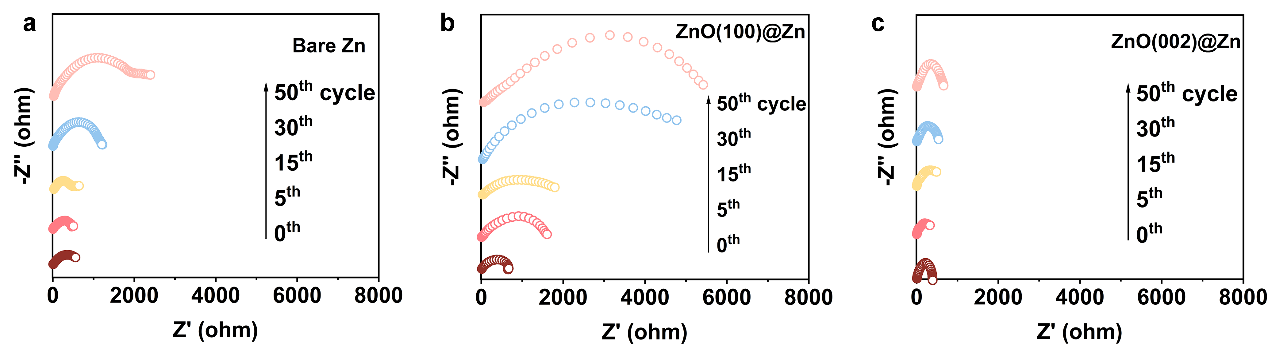


**Figure S27.** Nyquist plots of (a) bare Zn, (b) ZnO(100)@Zn and (c) ZnO(002)@Zn after different cycles.





**Figure S28.** XRD patterns of V_2_O_5_ cathode.


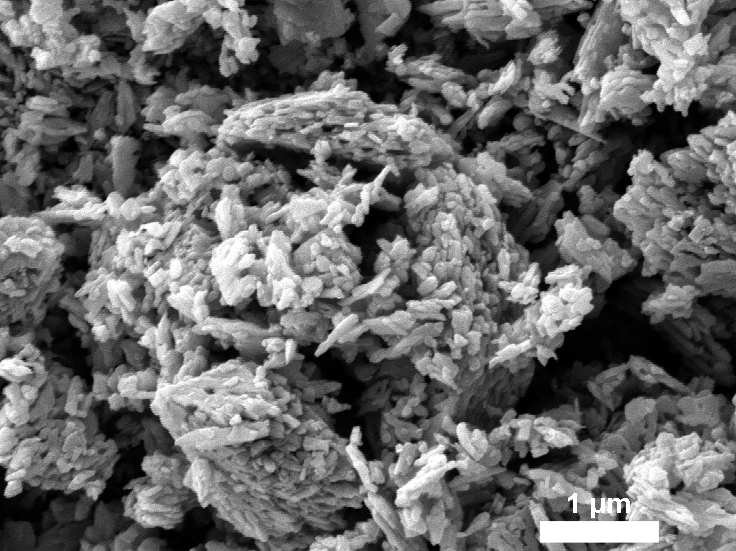


**Figure S29.** SEM image of V_2_O_5_ cathode material.


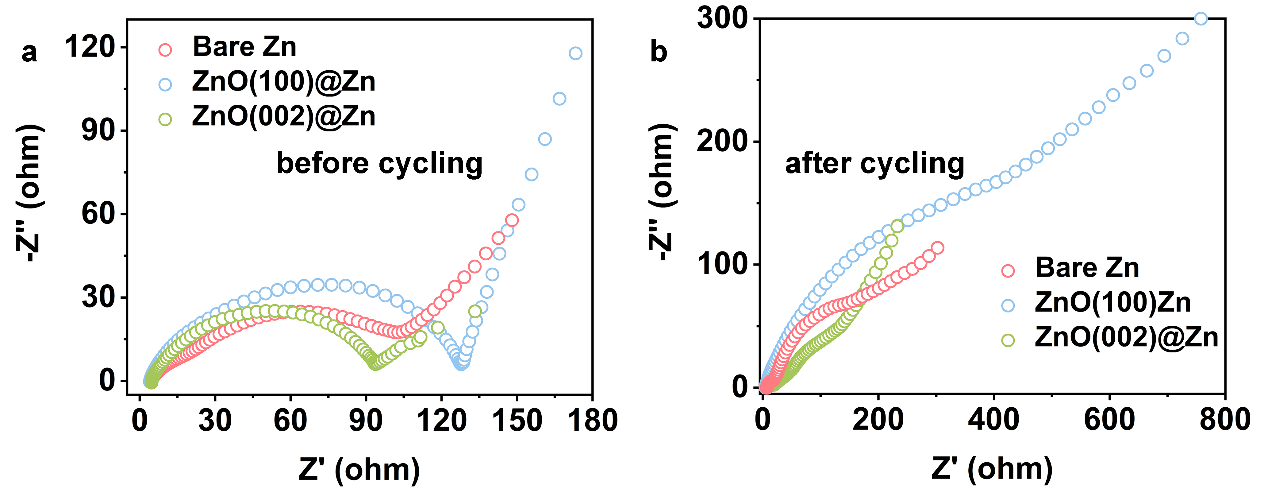


**Figure S30.** Nyquist plots of the Zn||V_2_O_5_ full cells with different anodes (a) before and (b) after cycling.


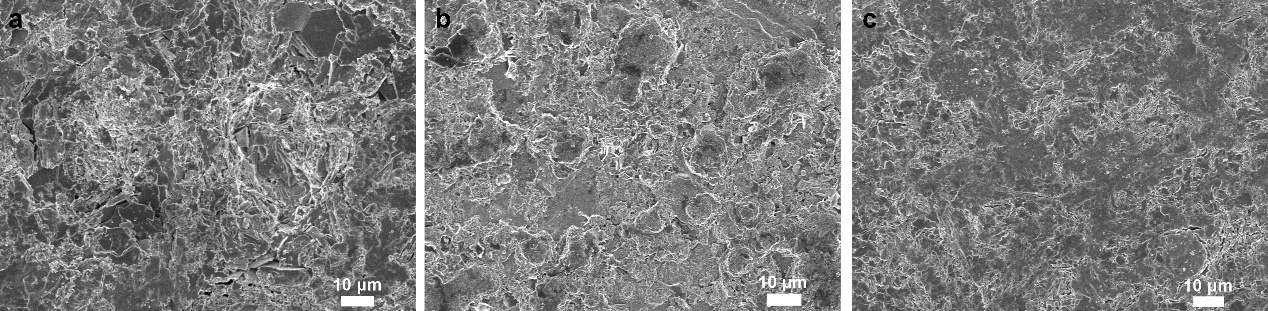


**Figure S31.** SEM images of (a) bare Zn, (b) ZnO(100)@Zn and (c) ZnO(002)@Zn after cycling in full cell.





**Figure S32.** XRD patterns of bare Zn, ZnO(100)@Zn and ZnO(002)@Zn anodes after 500 cycles of full cells. ZnO(002)@Zn exhibits a I_(002)/(101)_ ratio of 1.17, higher than bare Zn (0.80) and ZnO(101)@Zn (0.73), as well no by-product generation.





**Figure S33.** The cycling performance of ZnO(002)@Zn||V_2_O_5_ pouch cell at 0.05 A g^-1^.
